# Local Anæsthesia by Cocaine

**Published:** 1890-03

**Authors:** 


Local Anæsthesia by Cocaine.—M. Reclus regrets that cocaine has not come into more general use. Although it has been pronounced inefficacious and dangerous, the writer has successfully employed it in injections more than 700 times. He finds that *intradermic* injections alone are efficient; cutaneous applications are without effect, while applications to mucous surfaces may sometimes be employed for slight operations. Cocainized injections in the washing out of hydroceles and old hydrarthoses have given excellent results.

As interstitial injections alone possess much value in operations upon the integument, care must be taken that the injections penetrate the skin itself, instead of passing into the subcutaneous cellular tissue. If the parts are very thick it will be well to make several injections, one over the other. Under such conditions anæsthesia is always obtained, and the same is true of inflamed tissues, although this has been denied. The reporter has seen the effects obtained by these interstitial injections continue for an hour, and has been able to perform radical operations without inflicting pain. The bones alone seem insusceptible to this influence. In ano-rectal affections and in the painful operations necessitated by them, the employment of tampons saturated in a 2 per cent. solution and a series of interstitial injections, forming a circle, will secure complete anæsthesia, so that dilatation of the rectum even can be performed painlessly.

The reporter has made 700 injections without accident; he has carefully searched the literature of the subject and has found only four reports of death, and in these large quantities of cocaine were used, viz: 75 centigrm., 1.20 centigrm., 1.25 centigrm. and 1.50 centigrm. Below 75 centigrm. there have been no serious accidents. Such appearances as pallor of the face, shortness of breath, loquacity, tendency to syncope, etc., have been seen, but with ordinary doses, 20 centigrm. or more, accidents are very infrequent. Ordinarily 10 to 12 centigrm. are sufficient.—*Congres de Chirurgie*, 1889.

